# Supplementary material for: A Critical View of the Application of the APEX Software (Aqueous Photochemistry of Environmentally-Occurring Xenobiotics) to Predict Photoreaction Kinetics in Surface Freshwaters
Source: Molecules. 2019 Dec 18;25(1):9. doi: 10.3390/molecules25010009 (PMC7017383; doi:10.3390/molecules25010009)
Supplement: Supplementary file 1 [file molecules-25-00009-s001.zip › 3.2.4_gcc-4.4.0.zip!/3.2.4_gcc-4.4.0/3.2.4_gcc-4.4.0/doc/octave/html/interpreter/Displaying-Images.html]

Displaying Images - Untitled


Next: Representing Images,
Previous: Loading and Saving Images,
Up: Image Processing


---

### 31.2 Displaying Images

A natural part of image processing is visualization of an image.
The most basic function for this is the `imshow` function that
shows the image given in the first input argument. This function uses
an external program to show the image. If gnuplot 4.2 or later is
available it will be used to display the image, otherwise the
`display`, `xv`, or `xloadimage` program is used. The
actual program can be selected with the `image_viewer` function.

— Function File: **imshow** (im)  
— Function File: **imshow** (im, limits)  
— Function File: **imshow** (im, map)  
— Function File: **imshow** (rgb, ...)  
— Function File: **imshow** (filename)  
— Function File: **imshow** (..., string\_param1, value1, ...)  
> Display the image im, where im can be a 2-dimensional
> (gray-scale image) or a 3-dimensional (RGB image) matrix.
>
> If limits is a 2-element vector `[`low`,` high`]`,
> the image is shown using a display range between low and
> high. If an empty matrix is passed for limits, the
> display range is computed as the range between the minimal and the
> maximal value in the image.
>
> If map is a valid color map, the image will be shown as an indexed
> image using the supplied color map.
>
> If a file name is given instead of an image, the file will be read and
> shown.
>
> If given, the parameter string\_param1 has value
> value1. string\_param1 can be any of the following:
>
> "displayrange": value1 is the display range as described above.
>
>
>
>
> **See also:** image, imagesc, colormap, gray2ind, rgb2ind.

— Function File: **image** (img)  
— Function File: **image** (x, y, img)  
> Display a matrix as a color image. The elements of x are indices
> into the current colormap, and the colormap will be scaled so that the
> extremes of x are mapped to the extremes of the colormap.
>
> It first tries to use `gnuplot`, then `display` from
> `ImageMagick`, then `xv`, and then `xloadimage`.
> The actual program used can be changed using the `image_viewer`
> function.
>
> The axis values corresponding to the matrix elements are specified in
> x and y. If you're not using gnuplot 4.2 or later, these
> variables are ignored.
>
>
>
> **See also:** imshow, imagesc, colormap, image\_viewer.

— Function File: **imagesc** (a)  
— Function File: **imagesc** (x, y, a)  
— Function File: **imagesc** (..., limits)  
— Function File: **imagesc** (h, ...)  
— Function File: h = **imagesc** (...)  
> Display a scaled version of the matrix a as a color image. The
> colormap is scaled so that the entries of the matrix occupy the entire
> colormap. If limits = [lo, hi] are given, then that
> range is set to the 'clim' of the current axes.
>
> The axis values corresponding to the matrix elements are specified in
> x and y, either as pairs giving the minimum and maximum
> values for the respective axes, or as values for each row and column
> of the matrix a.
>
>
>
> **See also:** image, imshow, caxis.

— Function File: [fcn, default\_zoom] = **image\_viewer** (fcn, default\_zoom)  
> Change the program or function used for viewing images and return the
> previous values.
>
> When the `image` or `imshow` function is called it will
> launch an external program to display the image. The default behavior
> is to use gnuplot if the installed version supports image viewing,
> and otherwise try the programs `display`, `xv`, and
> `xloadimage`. Using this function it is possible to change that
> behavior.
>
> When called with one input argument images will be displayed by saving
> the image to a file and the system command command will be called
> to view the image. The command must be a string containing
> `%s` and possibly `%f`. The `%s` will be replaced by
> the filename of the image, and the `%f` will (if present) be
> replaced by the zoom factor given to the `image` function.
> For example,
>
> ```
>           image_viewer ("eog %s");
> ```
>
> changes the image viewer to the `eog` program.
>
> With two input arguments, images will be displayed by calling
> the function function\_handle. For example,
>
> ```
>           image_viewer (data, @my_image_viewer);
> ```
>
> sets the image viewer function to `my_image_viewer`. The image
> viewer function is called with
>
> ```
>           my_image_viewer (x, y, im, zoom, data)
> ```
>
> where x and y are the axis of the image, im is the image
> variable, and data is extra user-supplied data to be passed to
> the viewer function.
>
> With three input arguments it is possible to change the zooming.
> Some programs (like `xloadimage`) require the zoom factor to be
> between 0 and 100, and not 0 and 1 like Octave assumes. This is
> solved by setting the third argument to 100.
>
>
>
> **See also:** image, imshow.
